# Liver transplantation and primary liver cancer in porphyria

**DOI:** 10.1111/liv.15894

**Published:** 2024-03-08

**Authors:** Mattias Lissing, Bruce Wang, Staffan Wahlin

**Affiliations:** ^1^ Hepatology Division, Department of Upper GI Diseases Karolinska University Hospital Stockholm Sweden; ^2^ Department of Medicine, Huddinge Karolinska Institutet Stockholm Sweden; ^3^ Department of Medicine and Division of Gastroenterology University of California San Francisco San Francisco California USA

**Keywords:** acute porphyria, erythropoetic porphyria, liver neoplasms, liver transplantation, metabolic diseases, porphyria

## Abstract

The porphyrias are a heterogeneous group of metabolic disorders that result from defects in heme synthesis. The metabolic defects are present in all cells, but symptoms are mainly cutaneous or related to neuropathy. The porphyrias are highly relevant to hepatologists since patients can present with symptoms and complications that require liver transplantation (LT), and some porphyrias are associated with a high risk for primary liver cancer (PLC). Among the cutaneous porphyrias, erythropoietic protoporphyria (EPP) can lead to cholestatic liver failure where LT cures the liver disease but not the porphyria. In acute porphyria (AP), neurotoxic porphyrin precursors are produced in the liver and LT is a curative treatment option in patients with recurrent severe neuropathic attacks. Patients with AP, mainly acute intermittent porphyria, have a significantly increased risk for PLC that warrants surveillance and adequate follow‐up of high‐risk groups. LT is well established in both EPP with liver failure and AP with recurrent attacks, but most transplant centres have little porphyria experience and cooperation between transplant hepatologists, and porphyria experts is important in the often‐difficult decisions on timing and management of comorbid conditions.

**Abbreviations**
ADP: aminolevulinic acid dehydratase deficiency porphyria
AFP: alpha‐fetoprotein
AIP: acute intermittent porphyria
ALA: aminolevulinic acid
AP: acute porphyria
EPP: erythropoietic protoporphyria
ESRD: end stage renal disease
HCC: hepatocellular carcinoma
HCP: hereditary coproporphyria
HMBS: hydroxymethylbilane synthase
HSCT: haematopoietic stem cell transplantation
LT: liver transplantation
PBG: porphobilinogen
PLC: primary liver cancer
RNA: ribonucleic acid
TACE: transarterial chemoembolization
VP: variegate porphyria
XLEPP: x‐linked erythropoietic protoporphyria

**Key points**

- Liver transplantation is a curative treatment in acute porphyria and a life‐saving treatment for liver failure in erythropoietic protoporphyria.
- Haematopoietic stem cell transplantation is a curative option after liver transplantation in erythropoietic protoporphyria.
- Patients with a history of active acute intermittent porphyria are at high risk of primary liver cancer.
- Identified high‐risk groups should be included in surveillance programs for early detection of liver cancer.

## 1 INTRODUCTION

The porphyrias are eight rare genetic diseases caused by defects in the enzyme chain that produces heme.^1^ They are traditionally classified as either hepatic or erythropoietic depending on the primary site of heme precursor overproduction. Three porphyrias; acute intermittent porphyria (AIP), hereditary coproporphyria (HCP) and variegate porphyria (VP) are commonly labelled acute porphyrias (AP) since they can all present with episodic acute neurovisceral attacks.^2^ As the main metabolic defect is in the liver, they are also labelled acute hepatic porphyrias. In various ways, the liver is involved in all porphyrias. Some porphyrias are caused by processes in the liver,^3^ some can damage the liver, some can lead to primary liver cancer (PLC)^4^, ^5^ and some are treated by liver transplantation (LT).^6^, ^7^

LT in the treatment of metabolic diseases can be performed for secondary organ damage, for slowing or halting the progression of disease or for curing disease manifestations.^8^ In the porphyrias, erythropoietic protoporphyrias (EPP and XLEPP) can develop a severe cholestatic liver disease that is often fatal without a liver transplantation.^9^ In AP, some patients have severe recurrent attacks that do not respond to treatment and LT is a curative treatment option.^7^ Hence, the treatment aims for LT in porphyrias can be quite different.

Patients with AP are at risk of developing PLC.^5^, ^10^, ^11^, ^12^ The risk is related to age, disease activity and subtype of AP. Most patients with AP and PLC do not have other chronic liver diseases or liver cirrhosis. Regular surveillance for early detection is recommended and individuals with high risk should be prioritized.^4^

Here, we provide an overview of the current knowledge of LT as a treatment option in porphyria and PLC as a complication of porphyria.

## 2 LIVER TRANSPLANTATION IN ACUTE PORPHYRIA

The first LT in AP was performed in 1989 on a 7‐year‐old boy with aminolevulinic acid (ALA) dehydratase deficiency porphyria (ADP),^13^, ^14^ a very rare form of autosomal recessive AP. The transplantation was technically successful but did not cure the ADP phenotype.

There is now an extensive cumulative experience of LT in AIP, the most common AP. LT has proven to cure the phenotype and reverse some of the extrahepatic complications seen in severe AIP. Around 3%–8% of patients with symptomatic AIP develop recurrent attacks, defined as >3 acute attacks per year.^15^, ^16^ Recurrent attacks mainly affect AIP women in their 20s–40s. They suffer from severe pain, neuropathy, gastro‐intestinal symptoms, fatigue, mood and sleep disorders, and are frequently admitted to hospital.^17^ These patients rarely fully recover between attacks and often develop chronic symptoms that are intensified during new exacerbations. As a result, these patients often have low quality of life, impaired social interaction, high rates of depression, daily use of opioids for pain management and increased risk of long‐term sick leave and disability.^18^, ^19^, ^20^ Severe AP with recurrent attacks also imposes a significant financial burden on both patients and the health care system.^19^ Most LT has been performed in patients with AIP with recurrent attacks.

The first report of LT for a patient with AIP and recurrent attacks was published in 2004 by Soonawalla et al.^7^ This pioneer procedure assumed that hepatic over‐production of porphyrin precursors was the primary cause of the continuous attacks in AIP. The patient was a 19‐year‐old woman with more than two years of repeated acute attacks that led to 37 admissions with 197 days in hospital, disrupted studies and poor quality of life. After the LT, biochemical activity measured by urinary excretion of ALA and porphobilinogen (PBG) normalized within days and the patient experienced complete clinical recovery. In a later case series by Dowman et al, ten patients transplanted in the UK from 2002 to 2010 also reported complete recovery of AIP symptoms and had acceptable survival rates.^21^ Nine of the patients were female, all had normalized biochemical activity but two died during follow‐up. A high rate of hepatic artery thrombosis, a rare but serious complication in LT was seen in 4 (40%) of the patients and led to one re‐transplantation.^22^ Another publication described successful combined liver and kidney transplantation in two patients with end stage renal disease (ESRD) and recurrent attacks of AIP.^23^ A more recent publication summarized the results on 38 patients with AIP (34 (89%) women) from 12 European countries who were transplanted between 2002 and 2019.^6^ The 1‐ and 5‐year survival rates were 92% and 82%, similar to patients undergoing LT for other indications during the same period. Two patients were re‐transplanted and nine died during follow‐up. Neuropathy improved in most patients, but severe pre‐LT neuropathy was associated with worse outcomes. Patients with chronic kidney disease (CKD) in most cases did not improve renal function after LT. Hepatic artery thrombosis occurred in 4 (11%) of the patients and led to two re‐transplantations and the death of one patient.

So far, all reported LT for AP were performed in patients with AIP, with the exception of the ADP child in 1989^13^, ^14^ and one patient with VP in whom the transplant indication was alcoholic cirrhosis.^24^ One publication reported the results from domino‐liver transplantations using explanted AIP livers.^25^ It was assumed that since AIP generally has low clinical penetrance and AIP with recurrent attacks is almost exclusively seen in women of reproductive age, AIP livers from younger women could safely be transplanted to older men. The assumption was unfortunately not confirmed. Two male AIP liver recipients developed elevated ALA and PBG as well as clinically significant neuropathy after transplantation with AIP grafts.

LT thus offers a phenotypic cure for the most severe cases of AIP. The need for LT in these patients may be influenced by recent pharmaceutical developments. Givosiran, an RNA interference therapy, is approved for the treatment of frequent recurrent attacks of AIP.^26^ Givosiran reduces the frequency of attacks, and the need for heme‐infusion therapy, and improves the quality of life in many patients with severe forms of AP.^27^ Long‐term outcomes and real‐life studies of this relatively new treatment are promising but still limited.^28^ The introduction of givosiran will probably reduce the need for LT in AIP, but LT remains an important curative option when all other treatments fail.^29^

## 3 LIVER TRANSPLANTATION IN ERYTHROPOIETIC PROTOPORPHYRIA

EPP is the most common form of erythropoietic porphyria. The disease is characterized by overproduction and accumulation of protoporphyrin, the highly hydrophobic and photoactive substrate of the ferrochelatase enzyme.^9^ Protoporphyrin accumulation leads to painful sensitivity to sunlight in all patients and hepatobiliary disease in some patients. The excess protoporphyrin originates in the bone marrow, enters the circulation in red blood cells, is taken up by hepatocytes and is excreted via the biliary tract.

The liver disease associated with EPP results from abnormal accumulation of protoporphyrin in the liver. Between 25% and 33% of patients with EPP display some degree of liver damage.^30^, ^31^ Usually, the liver disease is mild and manifests only by slight deviations in liver function tests or mild histological abnormalities.^31^, ^32^ Gallstone disease thought to result mainly from protoporphyrin precipitation in the gallbladder, may occur even in childhood.^33^, ^34^, ^35^ Less than 5% of patients with EPP develop significant liver disease,^36^ though some studies have reported higher rates.^37^, ^38^ The liver damage is characterized by cholestasis due to protoporphyrin precipitation within hepatocytes and the intrahepatic biliary tree.^39^ In some patients, the course of the liver disease is insidious. More often, a rapidly progressive cholestatic liver failure characterizes the symptomatic debut. In most of these patients, cirrhosis is already present^30^ but in a few only some degree of fibrosis is seen.^40^, ^41^

The first report on successful LT in a patient with EPP came in 1980.^42^ Since then, LT has become an established life‐saving treatment for the minority of patients with EPP who develop severe liver disease. The two largest reported case series consisting of 20 LT in the US^43^ and 35 in Europe^41^ performed between 1979 and 2008 have provided a good comprehension of results and complications unique to EPP. The overall survival rate after LT among patients with EPP is similar to that of patients transplanted for other causes. The European series recorded a ten‐year survival rate of 66 percent and the US series a ten‐year survival rate of 47 percent.

Transplantation of the liver does not stop protoporphyrin overproduction and accumulation in the bone marrow. The photosensitivity is not cured, and the transplanted liver is at risk of also becoming injured by excess protoporphyrin.^44^ These features explain most of the complications to LT that are characteristic of EPP. Different rates of disease recurrence in the transplanted livers could explain the difference in survival rates reported between the European and US cohorts.

Four frequent problems have been identified in LT for EPP‐associated liver disease: disease recurrence in the graft, phototoxic tissue injuries from surgical lights, high frequencies of postoperative neuropathies and biliary tract complications. All these problems may potentially influence the short and long‐term results.

While liver transplantation is a lifesaving treatment for end‐stage EPP liver disease, haematopoietic stem cell transplantation (HSCT) cures all phenotypic manifestations.^45^ HSCT can prevent recurrent disease in the liver graft and sequential LT and HSCT should be considered, especially in paediatric liver recipients as the anticipated long lifespan makes graft recurrence likely. In view of the benign nature of uncomplicated EPP, it is not reasonable to use HSCT in the treatment of photosensitivity. Even if reduced conditioning is used, HSCT risks are significant. The role of HSCT in EPP is therefore intimately connected to liver disease.^46^

Ideally, patients who are prone to severe liver disease or who already display signs of liver disease are identified before LT is required. If surveillance programs can detect progressive liver disease associated with EPP, it may be possible to instead offer prophylactic therapy or curative HSCT.^46^ Until protoporphyric liver damage can reliably be recognized at an early stage, LT will remain an important option in EPP.

## 4 PRIMARY LIVER CANCER IN ACUTE PORPHYRIA

It has become increasingly evident that AP is associated with a significant risk of PLC. Patients with the highest and lowest risks have been identified but much work remains before individualized risk assessment is possible for the majority of the AP population.

An association between PLC and AP was first reported in 1984 by Lithner and Wetterberg who identified 11 patients with PLC in a cohort of 206 patients with AIP in the north of Sweden between 1960 and 1981.^47^ Since then, several AP cohort studies from the Nordic countries, France, Switzerland, Germany and the USA have confirmed this association (Table 1).^5^, ^10^, ^11^, ^12^, ^15^, ^34^, ^48^, ^49^, ^50^, ^51^, ^52^ The majority of PLC in patients with AP are hepatocellular carcinoma (HCC), but cholangiocarcinoma and mixed HCC‐ cholangiocarcinoma tumours have also been reported.^12^, ^53^, ^54^ Several cohorts only include patients with AIP and the number of reported patients with VP and HCP are overall low. All reported cases of PLC with APas the main risk factor have been in patients over the age of 50.

**TABLE 1:** Summary of cohort studies assessing the risk of primary liver cancer in acute porphyria.

Year	1st Author	Country	AP Cohort (*n*)	PLC (*n*)	Relative risk
1984	Lithner^47^	Sweden	206	11	N/A
1988	Kauppinen^11^	Finland	245	7	61
1999	Linet^51^	Sweden, Denmark	296	5	70
2000	Andant^48^	France	650	7	36
2009	Schneider‐Yin^34^	Switzerland	145	4	N/A
2011	Innala^49^	Sweden	180	22	64
2013	Elder^15^	Europe^a^	N/A	14	N/A
2013	Sardh^12^	Sweden	179	23	86
2015	Lang^50^	Germany	49	1	35
2017	Baravelli^10^	Norway	251	9	108
2020	Saberi^52^	USA	327	5	N/A
2022	Lissing^5^	Sweden	1244	83	38

Abbreviation: N/A, not applicable.

^a^ Among the 11 contributing countries only 4 reported new PLC cases; 11 were reported from Sweden and one each from the Netherlands, UK and Switzerland.

Assessments of PLC incidence in AP can be problematic due to several reasons. Symptomatic AP disease is most common in the 3rd to 4th decades of life while PLC diagnosis mainly occurs after the age of 60 when many patients are no longer under active follow‐up. PLC incidence in older patients with asymptomatic porphyria may therefore be underestimated. Even in large AP cohorts, PLC is a rare outcome and extensive follow‐up is needed to achieve statistical power for subgroup analysis. In the cohorts mentioned above, the number of patients who developed PLC was below 10 in each except for three overlapping Swedish studies.^5^, ^12^, ^49^ The definitions of symptomatic disease were based on subjective reports in several studies, which entails a risk of overestimating the proportion of symptomatic patients. Porphyrin precursor biochemistry data is scarcely reported in most studies. Lastly, only two of the studies are prospective and the composition regarding the mean age at baseline and the proportions of patients with VP and HCP differ significantly between studies.

### 4.1 HCC carcinogenesis in AP

AP‐associated HCC differs from HCC in non‐AP patients in several aspects. Most HCCs develop in the setting of long‐standing inflammation associated with an underlying chronic liver disease that leads to liver cirrhosis or advanced fibrosis. Less than 20% of all HCCs develop in non‐cirrhotic livers.^55^, ^56^ Male sex is an independent risk factor in non‐AP HCC, with a male‐to‐female ratio of 1.4–3.3.^57^ In AP, cirrhosis, high alcohol consumption or other underlying liver diseases are found only in a minority of patients with PLC, and women have a similar or somewhat higher risk than men.^12^

Little is known about the carcinogenic mechanisms involved in the development of HCC in AP, but the features mentioned above indicate an AP‐specific tumour development process. Several hypotheses have been proposed, including: (1) Impaired antioxidant levels due to heme deficiency,^58^ (2) a second somatic mutation in the genes controlling heme biosynthesis^54^, ^59^, ^60^ and (3) carcinogenic effects by ALA.^61^ Several studies support the hypothesis of ALA as an important mediator. ALA accumulates in the liver, blood and urine during and after attacks and can cause oxidative stress, DNA damage and is cytotoxic in vitro.^62^, ^63^, ^64^, ^65^ ALA levels are also increased in the liver in tyrosinemia type 1, which has been associated with a high risk of early liver cancer.^66^

### 4.2 Epidemiology of AP‐associated PLC

All studies in Table 1 report at least two‐digit relative risks, but estimates vary significantly, from 35 to 108 compared with the general population. It has been speculated that the high incidence of AP‐associated PLC found in Swedish studies might be related to the presence of the dominating *HMBS* founder gene variant (c.593G>A), found in approximately 2/3 of Swedish patients with AIP.^49^, ^67^ No support for this hypothesis has been published and two recent large studies from Norway and Sweden found no association between genotype and risk for PLC.^5^, ^10^ The marked variation in incidence and relative risk estimates is likely related to both differences in age distribution and the proportion of asymptomatic, biochemically inactive gene carriers vs patients with active disease included in the cohorts. Relative risk assessments are also affected by the PLC incidences in the reference populations, which vary with the prevalence of viral hepatitis and other liver diseases. The annual PLC incidence is low in the Nordic countries (Sweden; 5/100 000, Norway; 3.5/100 000) compared with France (12.9/100 000) and globally (9.5/100 000).^68^, ^69^ The Norwegian study by Baravelli et al. reported the highest relative risk but had a cohort with a median age of 53 in a country with low background PLC incidence while the French study by Andant et al. reported one of the lowest relative risks, but had a cohort with a younger median age of 41 years and higher PLC incidence in the reference population.^10^, ^48^ Similarly, the Swedish cohorts with the highest incidence also included patients with higher ages at baseline.^12^, ^49^ In a recently published large AP cohort study including 1244 patients with AP where 83 developed PLC, the relative risk (aHR) was 38 for patients with AP and 44 for patients with AIP. The annual incidence was .4/100 person‐years in the whole AP cohort and 1.4/100 person years after age 50.

A few studies have identified PLC as a more common cause of death among patients with AP compared to the general population. A registry‐based mortality study covering two municipalities in northern Sweden with a high prevalence of AIP reported that 9 of 33 (27%) patients with AIP died from PLC, compared to .2% in the non‐AIP‐population.^70^ Similarly, 54 (20%) patients died from PLC among the 265 patients with AP who died in Sweden from 1987 to 2015.^5^

### 4.3 AP–PLC risk factors

Several early studies indicated that patients with a history of symptomatic AP have a higher risk compared to asymptomatic or latent patients and that the proportion of women with AP and PLC was higher than expected compared to the reference populations.^5^, ^10^, ^49^ The definition of symptomatic AP varied between studies and many had no confirmatory laboratory testing. In the most recent studies, patients with a history of symptomatic AP, defined by hospital admissions and questionnaires, had a markedly increased PLC risk compared to those who were asymptomatic.^5^, ^10^ Few studies include data on historic biochemical activity but several report elevated porphyrin precursors at the time of PLC diagnosis. The most recent Swedish cohort study found that lifetime elevated peak PBG levels were strongly associated with PLC risk while none of the gene carriers with only normal PBG (*n* = 345) developed PLC. This was confirmed in a case–control study that also identified increasing ALA and PBG levels after age 65 as an indicator of increased risk of PLC.^71^ Some asymptomatic patients can be biochemically active with markedly elevated porphyrin precursor levels, now defined as asymptomatic high excreters.^72^ In older studies, these patients were likely defined as latent and it remains to be seen whether they have increased risk of PLC.

The incidence rate of PLC was higher among women than men in most studies but the marked difference in relative risk when compared to males and females in the general population is mainly due to the two to three‐fold male‐to‐female PLC risk increase in patients with non‐AP associated PLC. The risk difference between men and women with AP was insignificant when patients were stratified for biochemical activity.^5^ This suggests that higher AP disease activity, which is more common in women,^73^ rather than female sex per se, is associated with increased PLC risk.

Regarding the type of AP, most assessments of PLC risk are based on cohorts with a predominance of patients with AIP. Sporadic cases of PLC in patients with VP and HCP have been published,^11^, ^15^, ^74^, ^75^, ^76^, ^77^ but large VP or HCP cohort studies exploring PLC risk are lacking and specific risk estimates for these forms of AP are scarce. Patients with VP and HCP are often thought to have PLC risk similar to AIP. This assumption may be questionable as these diseases have considerable differences in genetics, clinical manifestation and biochemistry. In general, VP and HCP are less clinically active and have lower levels of ALA, which is regarded as the most likely porphyrin precursor to be involved in carcinogenesis. The French study by Andant et al. included 84 patients with HCP and 136 with VP^48^ and a study by the Swedish group included 56 (HCP) and 125 (VP) patients.^5^ Each study identified one HCP patient and one VP patient with PLC. Based on these small numbers and thereby low precision, risk estimates were clearly lower compared to AIP and not significantly different from those in the general population.^5^

### 4.4 PLC treatment and survival

Data on treatment, treatment‐related outcomes and survival in patients with AP and PLC is limited. In earlier studies, patients were generally diagnosed at a late, advanced stage and survival was poor. In the study Lithner in 1984, all 11 patients died from PLC, and none were eligible for resection.^47^ In the Finnish cohort study published in 1988, mean survival after PLC diagnosis was 7 months (range 5–12).^11^ Later studies reported better survival time and higher rates of patients given curative treatment options. Among the 22 patients with AP and PLC in the Swedish study by Sardh et al., a majority had initial treatments with curative intention, 9 (41%) were treated by resection, 3 (14%) with local ablation.^12^ In the recent study by Saberi et al., 80% of the patients had a liver resection and one was treated with transarterial chemoembolization (TACE). All patients were alive at the latest follow‐up with a mean follow‐up time of 79 months.^52^ This is likely related to improved treatment for HCC in general but also to increased awareness and surveillance of patients with AP. Innala et al. found that patients enrolled in a surveillance program had smaller tumour sizes relative to the non‐surveillance group. This resulted in a higher proportion of patients eligible for resection, 7/8 among those surveilled vs. 4/14 in the non‐surveillance group, and longer mean survival; 56 months in the surveillance group compared to 30 in the group with no surveillance.^49^ These results should however be interpreted with caution considering the high possibility of both lead‐time bias and selection bias due to the study design. Few studies have reported outcomes after treatment for PLC, but those that do have reported a relatively high frequency of recurrence ranging from 40% to 64%.^48^, ^49^, ^52^ To our knowledge, only two patients with AIP and PLC have received a liver transplant as treatment for PLC, both with favourable outcomes.^6^

### 4.5 HCC surveillance

The significant risk of PLC in AP has prompted recommendations for liver cancer surveillance by ultrasound imaging every 6–12 months from the age of 50 in patients with a history of symptomatic AP disease.^4^ Due to the previous lack of evidence, patients with biochemical activity who never had symptoms are not included in these recommendations. Based on recent studies it is recommended to include not only symptomatic patients but also patients with a history of biochemical activity. This in turn entails some form of regular porphyrin precursor monitoring. One aspect of surveillance recommendations is to identify high‐risk patients, who would most likely benefit from surveillance. Another is to identify low‐risk groups, in whom costly surveillance provides doubtful benefit but might cause anxiety and risk of unnecessary procedures due to false positive findings. Patients with AP < 50 years of age have low risk, but data on whether patients with asymptomatic AP and in particular those with VP and HCP have increased PLC risk that warrants surveillance are lacking.

The use of alpha‐fetoprotein (AFP) in HCC‐surveillance in general is debated^78^, ^79^ and the limited data on AFP from patients with AP and PLC is inconclusive. In the few studies that reported data on AFP levels at PLC diagnosis, the sensitivity was 0%, 11%, 28% and 100%,^12^, ^48^, ^49^, ^52^ which overall does not support the use of AFP in AP–PLC surveillance. Annual ultrasound surveillance has relatively low sensitivity for early PLC detection and might thus be ineffective in high‐risk groups.^80^ Guidelines on HCC surveillance in general recommends biannual surveillance in patients eligible for treatment if the annual incidence exceeds 1.5% (EASL guidelines) or .8% (AASLD guidelines) for patients with cirrhosis and lower for noncirrhotic patients.^78^, ^79^ The six‐month interval is related to the average HCC tumour progression rate which has not been described as different in patients with AP compared to other liver diseases. Hence, patients with AIP who are or have been biochemically and/or clinically active should undergo biannual ultrasound surveillance starting from age 50.

## 5 CONCLUSIONS AND FUTURE PERSPECTIVES

While porphyrias are genetic disorders of heme biosynthesis that affect all cells, many of its manifestations affect the liver. High‐PLC risk and the need for LT assessment are among several reasons why hepatological expertise often is essential in the management of porphyria patients. As we have highlighted in this review, AP is associated with a high risk of liver cancer that requires active surveillance and follow‐up. The risk is clearly increased among patients with AIP aged >50 years with a history of clinically or biochemically active disease while more studies are needed to assess the risk in VP and HCP. Several important questions remain regarding PLC in AP. The understanding of the disease‐specific carcinogenetic mechanisms involved in AP‐associated PLC is poor.^61^ Recent reports of biallelic loss of the HMBS gene within the liver tumour raise the possibility that heme biosynthesis genes may act as tumour suppressors in the liver.^54^, ^59^ Studies to explore this and the histopathological changes in liver tissue longitudinally and in relation to biochemical disease activity in patients with AP may shed light on the pathogenesis of PLC.

Liver transplantation can be performed from at least two different porphyria‐related indications; to cure porphyrin‐induced liver failure in EPP or as a cure for AP in patients with severe recurrent attacks. With the recent introduction of new drugs, the need for LT in AP will likely decrease but LT will continue to be an important option when all other therapeutical options have failed. With increased surveillance for PLC in patients with AP, LT will likely be used more frequently as curative treatment in these patients. Severe EPP‐induced liver disease remains a serious complication that is poorly understood. Early detection of EPP‐related liver disease is an important future research area but the current lack of ability to identify at‐risk patients early means that LT will remain the main treatment option for these patients in the foreseeable future.

## FUNDING INFORMATION

The authors have not received any grants for this article.

## CONFLICT OF INTEREST STATEMENT

The authors have no competing interests relating to this work.

## Data Availability

Data sharing is not applicable to this article as no new data were created in this review.
